# Pathogen Profiling in Reverse Total Shoulder Arthroplasty: Virulence Traits of Clinical Isolates Before and After Intraoperative Povidone–Iodine Irrigation

**DOI:** 10.3390/antibiotics15020129

**Published:** 2026-01-28

**Authors:** Enrico Bellato, Fabio Longo, Francesca Menotti, Rebecca Mariani, Lucrezia Massobrio, Valentina Bartolotti, Helena Villavicencio, Narcisa Mandras, Alessandro Bondi, Antonio Curtoni, Filippo Castoldi, Giuliana Banche, Valeria Allizond

**Affiliations:** 1 Department of Surgical Sciences, University of Torino, 10126 Turin, Italy; enrico.bellato@unito.it (E.B.); rebecca.mariani@edu.unito.it (R.M.); 2 Department of Public Health and Pediatrics, University of Torino, 10126 Turin, Italy; f.longo@unito.it (F.L.); francesca.menotti@unito.it (F.M.); lucrezia.massobrio@unito.it (L.M.); valentina.bartolotti@unito.it (V.B.); helena.villavicencio@unito.it (H.V.); narcisa.mandras@unito.it (N.M.); alessandro.bondi@unito.it (A.B.); antonio.curtoni@unito.it (A.C.); valeria.allizond@unito.it (V.A.)

**Keywords:** povidone-iodine, susceptibility pattern, biofilm production, coagulase-negative staphylococci, *Cutibacterium acnes*, disinfection protocol

## Abstract

*Background/Objectives*: Reverse total shoulder arthroplasty (RTSA), a commonly performed procedure in elderly patients with osteoarthritis, is frequently complicated by postoperative infections—primarily caused by *Cutibacterium acnes* and coagulase-negative staphylococci (CoNS)—which remain a major clinical challenge. While standard antiseptic skin protocols can reduce the bacterial load at the surgical site, they often fail to achieve complete eradication, particularly with *C. acnes*, a resident species of the shoulder microbiome. Recent evidence indicates that intraoperative povidone–iodine irrigation is effective in significantly decreasing microbial burden; however, a thorough characterization of the virulence factors of the isolated strains remains essential. *Methods*: A total of 187 clinical strains isolated immediately after RTSA were characterized with respect to their antibiotic resistance profiles and biofilm-forming capacity, and the impact of intraoperative povidone–iodine irrigation on the reduction in bacteria that express these virulence traits was evaluated. *Results*: Of the 120 *C. acnes* isolates, 97.67% were susceptible to the tested antimicrobial agents, while only 3.33% exhibited resistance, specifically to clindamycin. In contrast, 53% of CoNS isolates were classified as susceptible, whereas the remaining 47% demonstrated multidrug resistance. Biofilm production was detected in 24% (29/120) of *C. acnes* and 39% (25/64) of CoNS isolates, with a statistically significant reduction observed after irrigation only for *C. acnes*. No association was found between biofilm formation and clindamycin resistance in *C. acnes*, likely due to the low number of resistant isolates. Conversely, among CoNS, a correlation was observed, with the 17.2% of biofilm-producing strains also exhibiting resistance to antimicrobial agents. *Conclusions*: Notwithstanding the presence of these virulence factors, povidone–iodine irrigation proved effective in substantially reducing the number of bacterial isolates recovered at the surgical site without selecting for strains with enhanced pathogenicity. Notably, the majority of resistant bacteria were detected prior to intraoperative irrigation. This intraoperative procedure may be a key approach to reducing prosthetic joint infections frequently caused by more virulent pathogens, which are unlikely to be selected following this disinfection strategy.

## 1. Introduction

Reverse total shoulder arthroplasty (RTSA) is a widely used procedure to restore quality of life in elderly patients suffering from osteoarthritis and other shoulder conditions, such as rotator cuff tear arthropathy and complex proximal humeral fractures [1,2]. Due to its anatomical location, the shoulder area has a different microbiome compared to the knee and hip, with *Cutibacterium acnes* being the most prevalent, followed by Gram-positive cocci [3,4]. Although postoperative infections are relatively rare, prosthetic joint infections (PJIs) can occur, with reported incidence rates of approximately 1–3% across different studies [1,4,5]. The literature consistently identifies *C. acnes* as the most frequently isolated pathogen, followed by *Staphylococcus aureus* and coagulase-negative staphylococci (CoNS), particularly *S. epidermidis*, *S. hominis*, and *S. warneri* [3,6,7,8,9]. Despite the lower incidence compared with hip and knee arthroplasty, shoulder PJIs are associated with relevant clinical and antimicrobial management burdens, especially in infections caused by biofilm-forming and antimicrobial-resistant organisms [10]. Patient-related factors, including age, sex, comorbidities, and skin colonization by *C. acnes*, have been associated with an increased risk of shoulder PJIs [4,11], and standardized diagnostic and classification criteria are widely adopted in clinical practice for their accurate identification [12].

Antibiotic prophylaxis and standard antiseptic techniques are designed to reduce the bacterial presence and load in the skin surrounding the arthroplasty site and to prevent contamination of the surgical area during the operation [6,13,14]. However, a significant limitation is that none of these methods is able to completely eliminate microbial presence or the subsequent risk of infection. For instance, a double skin preparation of the shoulder using chlorhexidine gluconate followed by povidone–iodine has proven effective in reducing CoNS, but it has limited impact on *C. acnes* [14]. Likewise, a systematic review concluded that chlorhexidine gluconate is generally ineffective against *C. acnes*, whereas hydrogen peroxide demonstrated greater efficacy in reducing its presence [15].

In recent years, additional strategies have been introduced to further reduce the risk of infection during arthroplasty. Among these, intraoperative irrigation with povidone–iodine has demonstrated efficacy following knee and hip prosthesis implantation [16,17,18]. More recently, its application immediately after RTSA has also shown promising results, leading to a reduction in both aerobic and anaerobic flora, as well as in their specific bacterial counts [19]. Despite these findings, a comprehensive characterization of the recovered bacterial strains—particularly with respect to their antimicrobial susceptibility profiles, biofilm-forming capabilities, and key virulence factors—remains limited. Therefore, the aim of the present study was to assess the antibiotic susceptibility and biofilm formation ability of 187 clinical isolates. Additionally, we sought to investigate whether intraoperative irrigation with povidone–iodine is also associated with a reduction in bacteria that express these virulence characteristics.

## 2. Results

### 2.1. Determination of the Antimicrobial Susceptibility Pattern of the Bacterial Isolates

**Figure 1:**
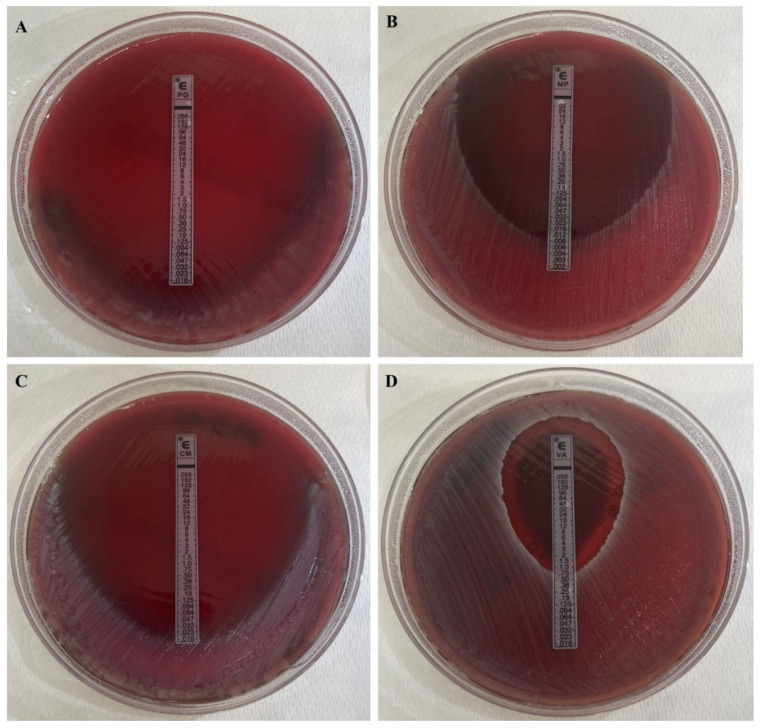
Representative images showing *C. acnes* isolate susceptible to Penicillin G (**A**), Meropenem (**B**), Clindamycin (**C**), and Vancomycin (**D**) on Schaedler agar plates, as determined by E-test.

**Figure 2:**
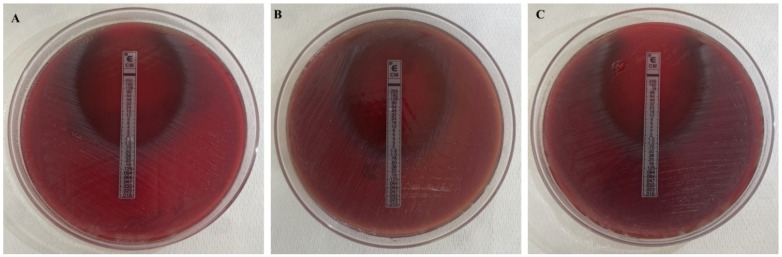
Representative images showing the *C. acnes* strains resistant to Clindamycin with different MIC values 0.75 µg/mL (**A**), 1 µg/mL (**B**) and 1.5 µg/mL (**C**) on Schaedler agar plates, as determined by E-test assay.

Among the 120 *C. acnes* isolates, 97.67% were generally susceptible to the tested antimicrobial agents—namely Penicillin G, Ampicillin, Meropenem, Clindamycin, and Vancomycin (Figure 1)—while only 3.33% exhibited a resistant profile (Table 1). Notably, only four strains—originating from the same patient—were resistant to Clindamycin, with MICs of 0.75 µg/mL (n = 1), 1.0 µg/mL (n = 2), and 1.5 µg/mL (n = 1) (Figure 2). Of these, three were isolated before the intraoperative irrigation and one after. No statistically significant differences in resistance profiles were observed between isolates collected before and after irrigation.

**Figure 3:**
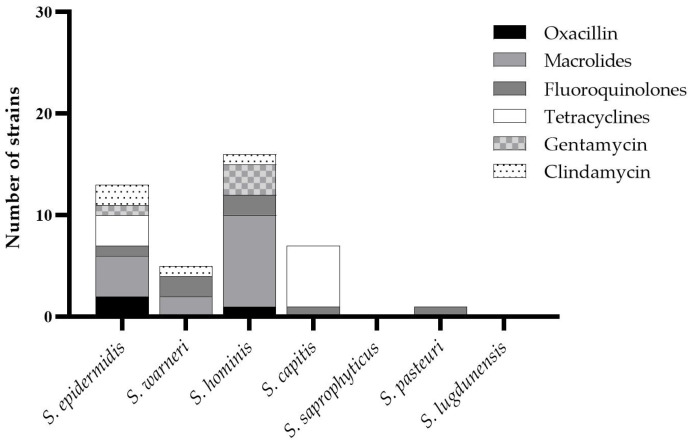
Distribution of the resistance pattern of CoNS towards the different antimicrobial agents assayed.

Among the CoNS isolates, 53% exhibited a fully susceptible profile, while the remaining 47% showed resistance to one or more antimicrobial agents. As illustrated in Figure 3, Methicillin resistance (MR)—defined by an Oxacillin MIC > 0.25 µg/mL—was detected in *S. epidermidis* (2 strains) and *S. hominis* (1 strain). Macrolide resistance, indicated by MICs ≥ 2 µg/mL for Erythromycin and/or Azithromycin, was observed in *S. hominis* (9 strains), *S. epidermidis* (4 strains), and *S. warneri* (2 strains). Fluoroquinolone resistance (MIC > 1 µg/mL for Moxifloxacin) was detected in *S. warneri* (2 strains), *S. hominis* (2 strains), *S. epidermidis* (1 strain), *S. capitis* (1 strain), and *S. pasteuri* (1 strain). Tetracycline resistance (MIC > 2 µg/mL) was identified in *S. capitis* (6 strains) and *S. epidermidis* (3 strains). Additionally, Gentamicin resistance (MIC > 4 µg/mL) was found in four strains—three *S. hominis* and one *S. epidermidis*—while resistance to Clindamycin (MIC > 2 µg/mL) was observed in two *S. epidermidis*, one *S. hominis*, and one *S. warneri* isolate. Notably, *S. aureus* and *E. coli* isolates were fully susceptible to all tested antimicrobial agents.

**Figure 4:**
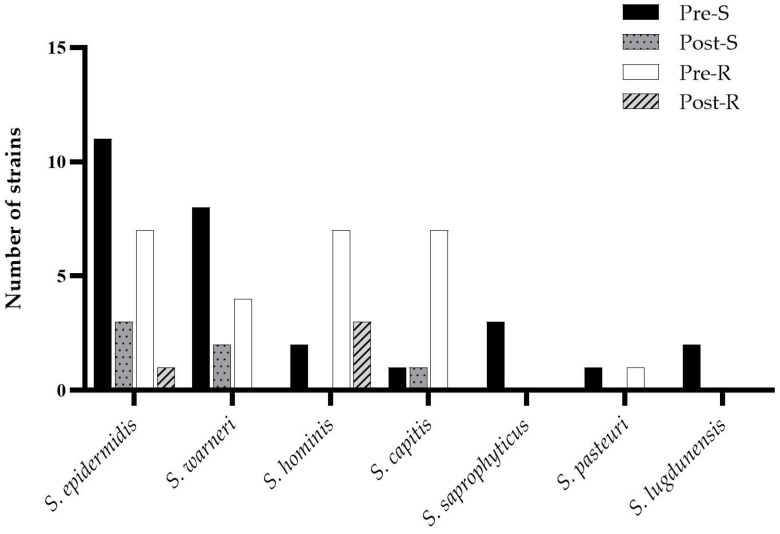
Subdivision of CoNS strains according to their sensitivity (S) or resistance (R) to the assayed antimicrobial drugs, before (pre) and after (post) the irrigation procedure.

As shown in Figure 4, most of the resistant strains were recovered prior to intraoperative irrigation. Although the procedure reduced the overall number of isolates, no statistically significant differences were observed in the distribution of susceptibility profiles between pre- and post-irrigation samples.

### 2.2. Determination of the Biofilm-Producing Capability of the Bacterial Isolates

**Figure 5:**
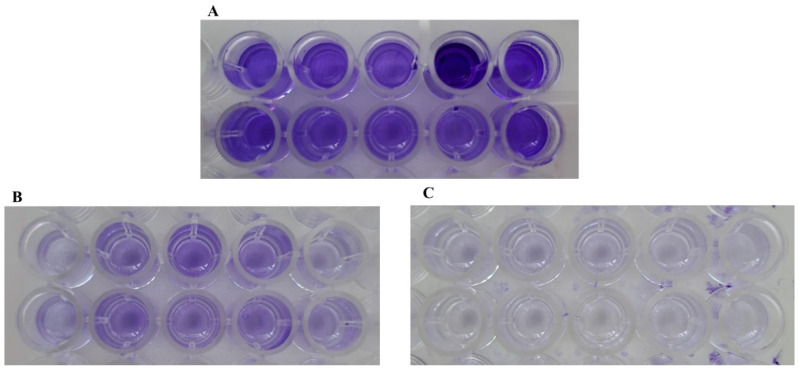
Representative images of crystal violet staining used to assess biofilm production: (**A**) strongly adherent, biofilm-producing strain; (**B**) weakly adherent strain; and (**C**) non-adherent, both considered non-biofilm-producing.

Finally, another key virulence factor known to negatively influence patient outcomes—the ability to produce biofilm—was assessed in vitro using the Tissue Culture Plate (TCP) assay for both anaerobic and aerobic isolates, as previously described. Figure 5 shows representative wells from the assay, illustrating a strong biofilm-producing strain (Figure 5A) in comparison to non-biofilm-producing strains (Figure 5B,C).

Among the *C. acnes* isolates, 29 out of 120 (24%) demonstrated biofilm-producing ability, classified as moderately or strongly adherent. The remaining 91 isolates (76%) were non-biofilm producers, displaying either a non-adherent or a weakly adherent profile. A higher number of antimicrobial-resistant and/or biofilm-forming isolates was recovered before intraoperative irrigation compared with post-irrigation samples. A comprehensive characterization of the *C. acnes* strains is provided in Table 2.

For the CoNS isolates, 39% exhibited biofilm production, while 61% were classified as non-biofilm-forming. Table 3 summarizes the distribution of moderate or strong biofilm-producing CoNS compared to weak or non-producers. In most species, the number of non-biofilm-producing strains exceeded that of biofilm producers. Exceptions included *S. capitis* and *S. pasteuri*, where biofilm producers were more prevalent, although both species were represented by a limited number of isolates. Moreover, also for these microorganisms, most isolates displayed a non-biofilm-forming profile compared with biofilm-producing isolates before intraoperative irrigation. Only a few biofilm-forming isolates were detected after irrigation, and these were represented by *S. epidermidis*, which was the most prevalent species among CoNS. A detailed species-level characterization of biofilm production before and after irrigation is reported in the Supplementary Materials (Table S1).

Regarding the other aerobic bacteria, the *S. aureus* isolate demonstrated biofilm production, while both *E. coli* isolates were not producers.

A statistically significant difference in biofilm production was observed among *C. acnes* strains prior to intraoperative irrigation (*p* = 0.0250), whereas no significant difference was found for CoNS (*p* = 0.5052), possibly reflecting the smaller number of CoNS isolates analyzed.

## 3. Discussion

The World Health Organization continues to update the list of antibiotic-resistant pathogens implicated in human infections and underscores the importance of addressing prosthetic joint infections (PJIs), including through the intraoperative use of povidone–iodine irrigation [20]. Several studies have supported the effectiveness of this approach in reducing infection rates following hip and knee arthroplasties [16,17,18]. More recently, its efficacy has also been demonstrated in RTSA, showing a reduction in both bacterial load and microbial diversity [19]. However, none of these studies conducted a full characterization of the isolated strains—when available—nor assessed whether the irrigation was associated with changes in their virulence factors. The aims of the present study were to determine the antimicrobial susceptibility patterns and biofilm production profiles of 187 clinical strains isolated from RTSA surgical sites, as well as to evaluate whether the intraoperative povidone–iodine irrigation could influence these virulence characteristics of the isolates.

*C. acnes* has gained increasing recognition in orthopedic surgery, not only in shoulder arthroplasty but also in procedures such as spine surgery. It is associated with PJIs, and the reported prevalence rates vary across the studies [21]. The current literature has described the susceptibility patterns of *C. acnes* to commonly used antimicrobial agents. Specifically, Baroudi et al. (2024) [21] reported high efficacy of penicillin, vancomycin, and cephalosporins. Núñez-Pereira et al. (2023) [13] highlighted the positive activity of rifamycin, while Ishida et al. (2008) [22] demonstrated that quinolones, tetracyclines, carbapenems, fusidic acid, and chloramphenicol were effective against this bacterium. Although considered a low-grade pathogen, *C. acnes* is generally susceptible to antimicrobials; however, recent studies have reported an increasing recovery of resistant strains. For instance, *C. acnes* has been reported as resistant to metronidazole, macrolides, trimethoprim/sulfamethoxazole, and clindamycin [21,23,24,25,26,27,28]. In the present study, we collected 120 intraoperative *C. acnes* isolates that were characterized for their antimicrobial susceptibility profiles. Our findings are in good agreement with those reported by other works as the only resistance observed—albeit in a small proportion of strains (~3%)—was to clindamycin, with MIC values ranging from 0.75 to 1.5 µg/mL [11,23,29,30]. Similarly, Broly et al. (2020) [31] reported clindamycin resistance in approximately 4% of *C. acnes* isolates, which were collected from both orthopedic surgery-related specimens and skin samples.

Additionally, aerobic Gram-positive—specifically CoNS and *S. aureus*—and Gram-negative bacteria, although rarely isolated, can serve as etiological agents of PJIs in the shoulder too. These microorganisms are well known for their resistance to a wide range of drugs [10,32]. In our study, we identified 64 CoNS, 1 *S. aureus* and 2 *E. coli* isolates directly from intraoperative swabs and tissue samples, all of which were characterized for their antimicrobial susceptibility testing profiles. Notably, 47% of the CoNS isolates exhibited resistance to one or more antimicrobial agents. In particular, 15/64 (23%) strains were resistant to macrolides, 9/64 (14%) to tetracyclines, 7/64 (11%) to fluoroquinolones, 4/64 (6%) to gentamicin and clindamycin, and 3/64 (5%) to methicillin, whereas the *S. aureus* and *E. coli* isolates were susceptible to all tested drugs. These findings are consistent with those reported by Coskun et al. (2024) [33], who showed that approximately 50% of isolates from orthopedic specimens were resistant to antimicrobials. In contrast, higher rates of methicillin-resistant CoNS and *S. aureus* were observed in their study [33]. Mitterer et al. (2022) [34] reported lower resistance rates (~17%) among the isolated microorganisms. However, consistent with our findings, *S. epidermidis* was the most prevalent CoNS species and exhibited resistance to multiple antibiotics, particularly tetracyclines, gentamicin, and fluoroquinolones [33,35]. Similarly, other authors [32] have also reported an increase in resistance to fluoroquinolones over recent years.

Despite the clinical relevance of PJIs, to the best of our knowledge, few studies have characterized the resistance profiles of CoNS species other than *S. epidermidis* and here *S. warneri*, *S. hominis*, and *S. capitis* were recovered from intraoperative clinical specimens. Among them, *S. hominis* was the most resistant species, with 83% of isolates showing resistance—particularly to macrolides, gentamicin, and fluoroquinolones; notably, one isolate was also identified as MR. In addition, 86% of *S. capitis* isolates were resistant to tetracyclines, while 14% of *S. warneri* isolates exhibited resistance to macrolides or fluoroquinolones. In a multicenter study, 215 CoNS strains causing PJIs were collected, with *S. hominis* identified as the most resistant species—particularly to ofloxacin, clindamycin, rifampicin, and trimethoprim/sulfamethoxazole—followed by *S. haemolyticus* [36]. The same authors also reported high resistance to fosfomycin in *S. capitis* (~67%) and *S. warneri* (~75%) [36]. De Vecchi et al. (2018) [37] and Ortega-Peña et al. (2019) [38] obtained a comparable pattern of resistance in CoNS—other than *S. epidermidis*.

Another challenging bacterial virulence factor is the ability to produce biofilm, as it enables pathogens to evade the host immune system and significantly reduces the effectiveness of infection treatment. In orthopedic surgery, this capability is well recognized in both *C. acnes* and CoNS [39,40,41]. However, most studies have focused on *C. acnes* isolates from the skin in the context of acne [42,43], despite its established role as a major component of the shoulder microbiome [44]. Eighty-five clinical strains of *C. acnes* were evaluated for their biofilm-forming capacity: the isolates were obtained from either skin swabs or deep surgical specimens, with the latter demonstrating a significantly higher level of biofilm production [44]. In another study, 14 strains from skin were subjected to biofilm production in vitro, and four of them displayed a greater biofilm formation [45]. Similarly, there is a paucity of literature analyzing in vitro biofilm production in CoNS clinical isolates specifically associated with orthopedic infections. For instance, Santos et al. (2022) [46] determined the production of biofilm by seven CoNS: *S. epidermidis* strains were strong producers, whereas variable profiles were noted for *S. saprophyticus*, *S. capitis* and *S. lugdunensis*. In a cross-sectional study, seventy-six strains were evaluated for biofilm production, and approximately 28% were identified as strong biofilm producers. Notably, these strains were not collected from deep surgical specimens [47]. In the present study, 29 out of 120 (24.2%) *C. acnes* isolates and 25 out of 64 (39.1%) CoNS isolates were identified as biofilm producers, exhibiting either strong or moderate adhesion. Among the CoNS, biofilm production was observed in 9 out of 22 *S. epidermidis*, 5 out of 9 *S. capitis*, and in both isolates of *S. lugdunensis* and *S. pasteuri*.

Regarding *C. acnes*, no association was observed between biofilm formation and clindamycin resistance. This finding is likely influenced by the limited number of clindamycin-resistant isolates detected in the present study but is in line with previous reports describing generally low rates of clindamycin resistance in *C. acnes* [23,31]. In contrast, for CoNS, such a correlation was evident: 11 out of 64 (17.2%) biofilm-forming strains exhibited antimicrobial resistance. Specifically, resistance was detected in two out of nine *S. epidermidis*, four out of five *S. capitis*, all four *S. hominis*, and one out of two *S. pasteuri* isolates. These findings are consistent with the existing literature, which shows that biofilm formation contributes to bacterial resistance to antibiotics in general [42,48,49,50], and particularly within orthopedic surgery [40,46,51,52]. In this context, Charalambous et al. (2022) [40] reported that CoNS, isolated from recurrent PJIs following failed hip and knee arthroplasties, exhibited increased antibiotic resistance associated with biofilm production. These observations are in line with the results presented here and with those of Koch et al. (2020) [51] and Tevell et al. (2017) [53], who specifically focused on *S. epidermidis* and *S. capitis*, respectively.

The last step was to evaluate whether the irrigation procedure contributed to the selection of bacterial strains with higher virulence—specifically, those exhibiting antimicrobial resistance and/or the ability to produce biofilm. Accordingly, the results provide a descriptive microbiological characterization of the recovered isolates, with specific reference to the limited number of CoNS strains. The results indicated that the higher number of strains isolated before irrigation, compared to those collected afterward, was associated with a significant proportion of antimicrobial-resistant and/or biofilm-producing isolates. Specifically, prior to intraoperative povidone–iodine irrigation, a total of 78/120 *C. acnes* and 53/64 CoNS were isolated. Among these, 3 *C. acnes* and 25 CoNS strains exhibited antimicrobial resistance, while 24 and 22 strains, respectively, demonstrated biofilm-forming capacity. In contrast, after the irrigation, only one *C. acnes* strain resistant to clindamycin was detected, along with five biofilm-forming strains. As for CoNS, five resistant and three biofilm-producing isolates were identified. It can be speculated that povidone–iodine, beyond its general antimicrobial activity, interferes with specific bacterial structures and functions, including membrane integrity, protein denaturation, and oxidative damage to cellular components. Experimental evidence suggests that povidone–iodine may also impair the synthesis and stability of extracellular polymeric substances, thereby affecting the early stages of biofilm development in staphylococci and *C. acnes* [41,54]. This mechanism may help eliminate pathogens at the surgical site before colonization of the implanted prosthesis occurs. These results confirm the efficacy of povidone–iodine irrigation in eliminating or reducing bacterial presence directly in the operative field without promoting the selection of more pathogenic strains. In this regard, specific clinical literature is currently lacking. However, some in vitro studies have shown that povidone–iodine is effective in reducing *C. acnes* counts and inhibiting biofilm formation by both staphylococci and *C. acnes* [55,56,57,58]. Additionally, the data obtained fit within a broader framework of environmental adaptation and phenotypic modulation. The variations observed in adhesion and biofilm assays may reflect microenvironment-dependent regulatory mechanisms and differences between planktonic and sessile bacterial populations, while also highlighting the inherent limitations of in vitro models in reproducing the complexity of the in vivo prosthetic joint environment. In this context, the observed phenotypic profiles may represent adaptive bacterial responses to local conditions rather than fixed traits. Future investigations integrating phenotypic analyses with genomic or transcriptomic approaches may help clarify bacterial adaptive responses and phenotypic modulation following exposure to intraoperative antiseptic strategies, complementing the in vitro phenotypic assays used in the present study.

## 4. Materials and Methods

### 4.1. Study Design and Clinical Isolate Collection

This observational microbiological investigation prospectively collected intraoperative clinical isolates from patients undergoing RTSA, as previously described [19]. Samples were collected before and after intraoperative irrigation, which was performed using a 0.35% sterile povidone–iodine (Betadine^®^, Meda Pharma S.p.A., Milan, Italy) solution applied for 3 min. All consecutive isolates recovered during the study period were included in the analysis. Microbial identification was performed by matrix-assisted laser desorption/ionization time-of-flight mass spectrometry (MALDI-TOF MS; Bruker Daltonics GmbH, Bremen, Germany). Clinical isolates were stored at −80 °C and subsequently characterized to detect potential multidrug-resistant bacteria by antimicrobial susceptibility testing and to assess biofilm-forming capability using crystal violet staining.

### 4.2. Antimicrobial Susceptibility Testing

For frozen *C. acnes* isolates, susceptibility testing was conducted using the E-test method for aerotolerant anaerobic bacteria according to European Committee on Antimicrobial Susceptibility Testing (EUCAST) guidelines. Inocula were prepared at a 1.0 McFarland turbidity standard (~3 × 10^8^ CFU/mL) and plated on CDC Anaerobe Agar with 5% sheep blood (BD, Milan, Italy), then incubated at 37 °C under anaerobic conditions for 48 h. The following antimicrobials were assayed: Penicillin G, Ampicillin, Clindamycin, Vancomycin and Meropenem (Biomerieux, Bagno a Ripoli, Florence, Italy).

Aerobic isolates recovered from frozen storage were sub-cultured on Columbia Agar with 5% sheep blood (BD, Milan, Italy) at 37 °C for 18–24 h prior to susceptibility testing with the MicroScan WalkAway system (Beckman Coulter s.r.l., Milan, Italy) using the Positive Combo 33 MicroScan Panel (i.e., Oxacillin, Penicillin, Vancomycin, Teicoplanin, Azithromycin, Clindamycin, Erythromycin, Ciprofloxacin, Levofloxacin, Moxifloxacin, Daptomycin, Trimethoprim/Sulfamethoxazole, Quinupristin/Dalfopristin, Linezolid, Tetracycline, Rifampicin, Gentamicin) for the aerobic Gram-positive bacteria (i.e., CoNS, *S. aureus*) and the Negative Combo-83 Panel (i.e., Ampicillin, Amoxicillin/Clavulanate, Piperacillin/Tazobactam, Piperacillin, Cefotaxime, Ceftazidime, Cefepime, Ertapenem, Imipenem, Meropenem, Aztreonam, Ciprofloxacin, Levofloxacin, Gentamicin, Amikacin, Tobramycin, Fosfomycin, Chloramphenicol, Colistin, Tigecycline, Trimethoprim/Sulfamethoxazole) for the aerobic Gram-negative bacteria (i.e., *E. coli*) according to the manufacturer’s instructions. All minimum inhibitory concentration (MIC) results were interpreted in accordance with the guidelines established by the EUCAST (https://www.eucast.org/bacteria/development-of-clinical-breakpoints-and-ecoffs/about-clinical-breakpoints/) (accessed on 22 September 2025).

### 4.3. Assessment of C. acnes Biofilm Formation

The ability of bacterial isolates to form biofilm was evaluated using the Tissue Culture Plate (TCP) assay [59]. In detail, *C. acnes* strains were cultured for 72 h at 37 °C in Fluid Thioglycollate Medium (FTM, Merck KGaA, Darmstadt, Germany) to obtain a bacterial suspension with a 1.0 McFarland density. These were then diluted 1:100 in FTM, supplemented with 2% glucose, resulting in a final concentration of approximately 10^6^ CFU/mL. Next, 200 µL of each diluted culture was dispensed into the wells of a 96-well microtiter plate and incubated at 37 °C for 3 and 5 days. After incubation, the supernatant was carefully removed, and each well was rinsed three times with sterile distilled water. The biofilm was fixed with 200 µL of 100% methanol (Carlo Erba Reagents s.r.l., Cornaredo, Milan, Italy) for 15 min. Following methanol removal, 200 µL of 1% crystal violet solution (Merck KGaA, Germany) was added and left for 15 min. Wells were then washed three times with distilled water, air-dried for 1 h, and treated with 200 µL of 95% ethanol to solubilize the stain. Optical density (OD) was measured at 595 nm using a VICTOR3™ microplate reader (PerkinElmer, Hopkinton, MA, USA) [59].

Each strain was tested in ten independent replicates for both incubation times, and mean OD values were calculated. Wells containing only FTM served as negative controls. The cut-off OD (ODc) was defined as three standard deviations above the mean OD of the control. Based on OD measurements, strains were classified according to their biofilm-forming capacity, in terms of adhesion (i.e., the ability of bacterial cells to attach to the substrate and initiate the early aggregate formation steps required for subsequent biofilm development), as reported in Table 4.

### 4.4. Assessment of Aerobic Bacteria Biofilm Formation

Biofilm formation was assessed for all aerobic Gram-positive and Gram-negative isolates through the TCP assay. For inoculum preparation, each strain was cultured in Tryptic Soy Broth (TSB, Merck KGaA, Germany) at 37 °C for 18–24 h. Bacterial cells were then suspended in 0.9% sterile NaCl to achieve a turbidity equivalent to a 0.5 McFarland standard and subsequently diluted 1:100 in TSB supplemented with 2% glucose to obtain a final concentration of approximately 10^6^ CFU/mL. A volume of 200 µL of each bacterial suspension was transferred into individual wells of a 96-well microtiter plate, followed by incubation at 37 °C for 24 h. Reference strain *S. epidermidis* ATCC 12228 strain was used as a negative control. After incubation, the supernatant was carefully removed, and each well was rinsed three times with sterile distilled water. Next, 200 µL of 100% methanol was added. After 15 min, wells were then emptied and stained with 1% crystal violet solution (Merck KGaA, Germany) for 15 min. Plates were washed again three times with sterile distilled water, air-dried for 1 h, and treated with 200 µL of 95% ethanol (Carlo Erba Reagents s.r.l., Italy) to solubilize the dye [59].

Optical density (OD) was measured at 595 nm using a VICTOR3™ microplate reader (PerkinElmer, USA). Each experiment was performed in ten independent replicates per strain, and average OD values were calculated. The cut-off OD (ODc) and biofilm production classification were determined as described above and in Table 4.

### 4.5. Statistical Analysis

Fisher’s exact test was used to evaluate differences in virulence factors of the strains before and after intraoperative povidone–iodine irrigation. A *p*-value < 0.05 was considered statistically significant.

## 5. Conclusions

The shoulder microbiome is distinct, with *C. acnes* as its predominant component, followed by CoNS—both of which are also the most common pathogens implicated in shoulder PJIs. Intraoperative irrigation with povidone–iodine has been shown to effectively reduce microbial burden in the surgical field. However, the present characterization of a substantial number of strains isolated immediately after the RTSA approach revealed that these bacteria possess virulence factors that may negatively influence patient outcomes following prosthesis implantation. Indeed, a substantial proportion of *C. acnes* and CoNS isolates were resistant to antimicrobial agents, and many also exhibited a biofilm-forming ability too. Despite these virulence traits, intraoperative povidone–iodine irrigation was associated with a reduction in the number of isolates recovered after irrigation, without an apparent enrichment of antimicrobial-resistant and/or biofilm-forming phenotypes. However, further studies are needed to clarify the direct effects of povidone–iodine and other disinfectants on both anaerobic and aerobic bacteria, in order to determine the most appropriate intraoperative irrigation strategy for minimizing the risk of PJIs in patients undergoing arthroplasty—where both external and internal factors may significantly influence clinical outcomes.

## Figures and Tables

**Table 1:** Susceptibility pattern of *C. acnes* strains (n = 120) towards different antimicrobial agents, reported by MIC values, µg/mL, number of isolates, and percentages.

Antimicrobial Agent	MIC Values (µg/mL)	Number of Isolates (%)
Penicillin G	≤0.016	101 (84%)
	0.012–0.047	14 (12%)
	0.06	5 (4%)
Ampicillin	≤0.016	98 (82%)
	0.023–0.047	10 (8%)
	0.06–0.125	10 (8%)
Meropenem	0.002–0.008	70 (58%)
	0.012–0.047	34 (28%)
	0.064	13 (11%)
	0.125	3 (3%)
Vancomycin	≤0.064	23 (19%)
	0.125–0.38	74 (62%)
	0.5–0.75	22 (18%)
	1.5	1 (1%)
Clindamycin	≤0.016	68 (57%)
	0.023–0.047	34 (28%)
	0.06–0.19	15 (12.5%)
	≤1.5	4 (3.33%)

**Table 2:** Summary of the biofilm producer and non-producer *C. acnes* isolates (as number of strains and percentage), before and after the povidone–iodine irrigation, reported by adhesion type.

*C. acnes* (n = 120)	Type of Adhesion	PRE	POST	Fisher’s Exact Test
BF (n = 29)	Strongly	7	1	*p* = 0.0250
	Moderate	17	4	
	Total	24 (20%)	5 (4%)	
NBF (n = 91)	Weakly	20	12	
	Non-adherent	33	26	
	Total	53 (44%)	38 (32%)	

Abbreviation: Biofilm-forming: BF; Not biofilm-forming: NBF; Before povidone–iodine irrigation: PRE; After povidone–iodine irrigation: POST.

**Table 3:** Summary of the biofilm producer and non-producer CoNS isolates (as number of strains and percentage), before and after the povidone–iodine irrigation, reported by adhesion type.

*CoNS* (n = 64)	Type of Adhesion	PRE	POST	Fisher’s Exact Test
BF (n = 25)	Strongly	5	2	*p* = 0.5052
	Moderate	17	1	
	Total	22 (34%)	3 (5%)	
NBF (n = 39)	Weakly	25	4	
	Non-adherent	6	4	
	Total	31 (48%)	8 (13%)	

Abbreviation: Biofilm-forming: BF; Not biofilm-forming: NBF; Before povidone–iodine irrigation: PRE; After povidone–iodine irrigation: POST.

**Table 4:** Adhesion profile and classification of biofilm production based on optical density (OD) measurements at 595 nm.

OD Value	Adhesion	Biofilm Production
OD ≤ ODc	Not adherent	Biofilm non-producer
ODc < OD ≤ 2 × ODc	Weakly adherent	
2 × ODc < OD ≤ 4 × ODc	Moderately adherent	Biofilm producer
4 × ODc < OD	Strongly adherent	

Abbreviations: optical density, OD; optical density cut-off value, ODc.

## Data Availability

All the data generated or analyzed during this study are included in this published article.

## Supplementary Materials

The following supporting information can be downloaded at: https://www.mdpi.com/article/10.3390/antibiotics15020129/s1, Table S1: Summary of the biofilm producer and non-producer CoNS isolates, before and after the povidone–iodine irrigation, reported by species.
