# Supplementary material for: Pathogen Profiling in Reverse Total Shoulder Arthroplasty: Virulence Traits of Clinical Isolates Before and After Intraoperative Povidone–Iodine Irrigation
Source: Antibiotics (Basel). 2026 Jan 28;15(2):129. doi: 10.3390/antibiotics15020129 (PMC12937305; doi:10.3390/antibiotics15020129)
Supplement: Supplementary file 1 [file antibiotics-15-00129-s001.zip › antibiotics-4071887-supplementary.pdf]

Table S1. Summary of the biofilm producer and not producer CoNS isolates, before and after the povidone-iodine irrigation, reported by species.

CoNS (n=64)		Type of adhesion	PRE	POST	Fisher's exact test
S. epidermidis (n=22)	BF (n=9)	strongly	2	1	*p>0.9999*
		moderate	5	1	
		total	**7**	**2**	
	NBF (n=13)	weakly	10	2	
		non-adherent	1	0	
		total	**11**	**2**	
S. warneri (n=14)	BF (n=2)	strongly	0	0	*p>0.9999*
		moderate	2	0	
		total	**2**	**0**	
	NBF (n=12)	weakly	8	1	
		non-adherent	2	1	
		total	**10**	**2**	
S. hominis (n=12)	BF (n=4)	strongly	1	0	*p= 0.4909*
		moderate	3	0	
		total	**4**	**0**	
	NBF (n=8)	weakly	4	0	
		non-adherent	1	3	
		total	**5**	**3**	
S. capitis (n=9)	BF (n=5)	strongly	1	0	*p= 0.4444*
		moderate	4	0	
		total	**5**	**0**	
	NBF (n=4)	weakly	2	1	
		non-adherent	1	0	
		total	**3**	**1**	
S. saprophyticus (n=3)	BF (n=1)	strongly	0	0	*p>0.9999*
		moderate	1	0	
		total	**1**	**0**	
	NBF (n=2)	weakly	1	0	
		non-adherent	1	0	
		total	**2**	**0**	
S. lugdunensis (n=2)	BF (n=2)	strongly	0	0	*p>0.9999*
		moderate	2	0	
		total	**2**	**0**	
	NBF (n=0)	weakly	0	0	
		non-adherent	0	0	
		total	**0**	**0**	
S. pasteuri (n=2)	BF (n=2)	strongly	1	1	*p>0.9999*
		moderate	0	0	
		total	**1**	**1**	
	NBF (n=0)	weakly	0	0	
		non-adherent	0	0	
		total	**0**	**0**	

Abbreviation: Biofilm forming: BF; Not biofilm forming: NBF; Before povidone-iodine irrigation: PRE; After povidone-iodine irrigation: POST.
